# Nose-to-Brain Delivery

**DOI:** 10.3390/pharmaceutics12020138

**Published:** 2020-02-06

**Authors:** Paolo Giunchedi, Elisabetta Gavini, Maria Cristina Bonferoni

**Affiliations:** 1 Department of Chemistry and Pharmacy, University of Sassari, 07100 Sassari, Italy; eligav@uniss.it; 2 Department of Drug Sciences, University of Pavia, 27100 Pavia, Italy; cbonferoni@unipv.it

**Keywords:** nasal route, brain targeting, nanocarriers, nanoemulsions, penetration enhancers, transmucosal delivery

## Abstract

Nose-to-brain delivery represents a big challenge. In fact there is a large number of neurological diseases that require therapies in which the drug must reach the brain, avoiding the difficulties due to the blood–brain barrier (BBB) and the problems connected with systemic administration, such as drug bioavailability and side-effects. For these reasons the development of nasal formulations able to deliver the drug directly into the brain is of increasing importance. This Editorial regards the contributions present in the Special Issue “Nose-to-Brain Delivery”.

The blood–brain barrier (BBB) separates the central nervous system (CNS) from the systemic circulation. The barrier characteristics of BBB depend on the properties of the brain endothelial cells that constitute the walls of the blood vessels. There are many neurological diseases such as neurological infections, Parkinson’s disease, Alzheimer’s disease, multiple sclerosis, age-related neurodegenerative diseases, and cerebral ischemia, that require a therapy in which the drug must reach the brain. Furthermore, many of these diseases need chronic therapies. Drug targeting to the brain represents a big challenge because many of these drugs do not cross BBB. Therefore, many efforts must be done for the design of strategies to solve this problem. The use of the nose-to-brain delivery route is an important and noninvasive method of drug delivery to bypass the BBB. In fact, it is well-known that there is an intranasal direct anatomical connection between the nasal cavity and the CNS, which suggests the development of nasal formulations for brain targeting of drugs. 

Different strategies have been developed for nose-to-brain drug delivery, that also involve nanomedicine with different kinds of nanocarriers: polymeric nanoparticles, nano-emulsions, dendrimers, and nano-micelles. These systems can also be bioadhesive. The development of new nasal systems represents a great challenge in the field of controlled drug targeting and delivery.

This Special Issue was intended to highlight the current progress in the use of the nasal route for brain targeting. A series of research articles and reviews are here present and offer a summary of researches by different groups, making a significant contribution in the field.

The research section includes five papers. Bonferoni et al. proposed a chitosan salt of chitosan and oleic acid, chitosan oleate, as amphiphilic polymer to obtain polymeric nano-micelles loaded with geraniol, an acyclic monoterpene [1]. Geraniol is abundant in essential oils extracted from aromatic plants and it has anticancer and neuroprotective properties. The nano-emulsions were in vitro characterized: the formulation based on 2% (w/w) of chitosan oleate showed viscosity values compatible with oral and nasal administrations to rats, and mean diameter of the dispersed phase of about 800 nm. The results of in vivo tests carried out on rats showed that the oral administration of this formulation induced not only a great increase of geraniol bioavailability, but also an enhancement of its aptitude to target the CNS from the bloodstream in comparison to formulations constituted by coarse geraniol emulsion. Moreover, appreciable geraniol amounts were detected in the CNS following the nasal administration of relatively low doses of the chitosan oleate emulsion of geraniol.

The work of Espinoza et al. is a clear example of the use of the nose-to-brain delivery, to overcome the problems connected to the oral drug administration [2]. Donepezil is a drug that increases cholinergic transmission, decreases neural toxicity caused by β-amyloid, decreases glutamate-induced neurotoxicity. This drug is administered orally for the therapy of Alzheimer’s disease. However, the drug is characterized by a low bioavailability in the brain owing to its poor ability to penetrate the BBB and the oral administration presents additional problems such as first-pass metabolism and adverse effects in the gastrointestinal system. The work demonstrated that the intranasal route represents an alternative to the oral administration of this drug. Drug loaded nano-emulsions containing Pluronic F-127 were prepared. The characterization of the formulations were made studying parameters such as physical properties, stability, in vitro release profile and ex vivo permeation using porcine nasal mucosa. In vivo tolerance of the developed formulations was evaluated using pigs. The results obtained showed that the use of Pluronic F-127 is effective in improving the characteristics of the formulation for the nasal administration. The histopathological analysis of porcine nasal mucosa showed that there was no infiltration of inflammatory cells nor significant changes.

Tzeyung et al. made an interesting study whose aim was the development and evaluation of rotigotine-loaded chitosan nanoparticles for nose-to-brain delivery [3]. Rotigotine is a non-ergoline dopamine D1/D2/D3 agonist that is used in the therapy of Parkinson’s disease. The nanoparticles were prepared by the ionic gelation method and were in vitro characterized. Ex vivo permeation studies were carried out using goat nasal mucosa. The effect of the application of the nanoparticles on the structure of nasal mucosa was determined by a histopathological study of untreated goat mucosa and goat mucosa subjected to 24 h of the permeation test. The results obtained from in vitro release tests showed that the nanoparticles are characterized by a sustained drug release. The histopathological examination of nasal mucosa showed no toxicity or structural damages. The results obtained by this work show that rotigotine loaded chitosan nanoparticles represent a potential formulation for nose-to-brain delivery to be used in the therapy of Parkinson’s disease.

Rassu et al. described the preparation of chitosan nanoparticles loaded with Genistein, an isoflavonoid phytoestrogen that presents antioxidant and neuroprotective activities, but also characterized by low water solubility, rapid metabolism and consequent poor oral bioavailability that limit its clinical application [4]. Drug loaded chitosan nanoparticles were prepared by the ionic gelation method, with optimization of different parameters. The prepared nanoparticles were in vitro characterized, showing that particles of about 75 nm were obtained, with small polydispersity index and very high drug loading efficiency (96%). Ex-vivo permeability tests of drug loaded nanoparticles was carried out using swine nasal mucosa compared to a drug suspension in phosphate buffer pH 6.5. Cytotoxicity of the nanoparticles was tested on PC12 cells, as neuronal model cells. These tests showed that nanoparticles are able to promote drug diffusion through the nasal mucosa and do not show significant changes in terms of cell viability and apoptotic events on PC12 cells.

Ladel et al.’s work regards the therapeutic use of antibodies for the treatment of neurological diseases [5]. The blood-brain barrier (BBB) is a highly selective barrier and limits also the use of antibodies for the therapy of diseases of the central nervous system (CNS). The neonatal Fc receptor (FcRn) has a fundamental role in the transepithelial transcytosis of immunoglobulin G. The scope of the research was the determination of the presence of FcRn in (ex vivo) porcine olfactory mucosa and the evaluation of its role in nose-to-brain drug delivery. FcRn was found in epithelial and basal cells of the olfactory epithelium, in glands, cavernous bodies and blood vessels. Allogenic porcine IgGs were found time-dependently in the lamina propria and along axonal bundles; small amounts of xenogenic human IgGs were detected. Interestingly, lymphoid follicles were spared from allogenic IgGs. The conclusions of the authors were that Fc-mediated transport of IgG across the nasal epithelial barrier may have significant potential for intranasal delivery, but the relevance of immune interaction in lymphoid follicles must be clarified to avoid immunogenicity.

Rinaldi et al. made an interesting study on chitosan glutamate niosomes, non-ionic surfactant nanoscale-vesicles, which are proposed as formulations for nose-to-brain drug delivery [6]. The study describes the preparation and characterization of both “blank” and drug loaded chitosan niosomes. The model drug that has been chosen is pentamidine which is an example of old drug (antiprotozoal discovered in 1938 for treatment of *Pneumocystis jirovecii* pneumonia) characterized by novel properties: recently it was found that this drug has anti-inflammatory and neuroprotective activities in Alzheimer’s disease. Mucoadhesive properties and stability testing in various environments were evaluated. The progression of Alzheimer’s disease can be delayed by the anti-inflammatory and antiproliferative properties of pentamidine. However, this drug has problems of low bioavailability and high hepatotoxicity that limit its oral administration. For these reasons it is important the preparation of a formulation able to give a nose-to-brain drug delivery. Niosomes were prepared using the thin film hydration technique. In vitro characterization and interaction studies with mucin were made by performing fluorescence turbidity measurements using luminescence spectrometer. The results of this study showed that the niosomes prepared are suitable for nasal administration and have potentialities as formulations containing pentamidine.

The review-section includes seven papers. These reviews include the more recent developments in the area of nose-to-brain delivery, relevant literature is present in their references, they add new contributions in the area. Musumeci et al. wrote an interesting review regarding the use of polymeric nanoparticles for the nose-to-brain delivery in the therapy of epilepsy [7]. Epilepsy is one of the most common global neurological diseases. Its therapy is chronic and usually consists in oral or intravenous administrations of antiepileptic drugs which present some limits, connected to problems of low-bioavailability, side-effects, first-pass metabolism. The nose-to-brain drug delivery can be, therefore, an important way to overcome these problems. In the first part of the review the pathophysiological characteristics of epilepsy, the diagnostic/therapeutic problems are clearly described and the main antiepileptic drugs are reviewed with their limits. There is also a comprehensive overview of the “state-of-the-art” in the field of drug formulation for nose-to-brain delivery, putting in evidence the importance of the so-called “3N” rule: nasal route, nanomedicine, and neurotherapy.

Bonferoni et al. wrote a review regarding a topic of increasing interest: nanoemulsions applied to the nose-to brain delivery [8]. Nanoemulsions are oil-in-water (O/W) or water-in-oil (W/O) dispersions of two immiscible liquids stabilized using appropriate surfactant(s), with a mean droplet diameter of about 100 nm. Nanoemulsions have droplets of a small size and therefore they are characterized by a higher surface area with respect to other formulations. A general overview of nanoemulsions for nose-to-brain delivery is made by the authors with a useful classification of this kind of formulations, based on the loaded drug and on the therapeutic purposes. The formulations used in the intranasal administrations of drugs are always O/W emulsions. The aim of this review is to put in evidence which is the strength of nanoemulsions in the nose-to-brain drug delivery. As demonstrated by the reported literature, intranasal administrations of nanoemulsions often lead to better results, also with respect to intravenous administrations. These good results can be explained by mechanisms of transcytosis/endocytosis of the nanodroplets by the brain endothelial cells. Furthermore, mucoadhesive polymers can be added in their composition to slow down nasal clearance.

Teleanu et al.’s review regards in general the use of nanotechnologies to obtain formulations suitable for nose-to-brain delivery [9]. It is reported an overview of nanotechnology approaches for crossing the blood-brain barrier that involve different kinds of nanoparticles: polymeric nanoparticles, liposomes, dendrimers, micelles, gold nanoparticles, silica nanoparticles, and carbon nanotubes. The authors put in evidence that nanotechnology-based approaches are intensively studied at the moment to overcome BBB and deliver the appropriate amount of drug to the specific brain site.

Gänger and Schindowski made a review on architecture, physico-chemical characteristics and mucociliary clearance of the nasal olfactory mucosa [10]. The authors review the “state-of-the-art” of the knowledge of the characteristics of the nasal and olfactory mucosa needed for a rational design of intranasal formulations. The information present in the literature reported in this paper are very important for every researcher that wants to design suitable formulations for nose-to-brain drug delivery. The authors put in evidence that to design and prepare N2B delivery systems, one has to understand the unique structure of the olfactory region. Avoiding immunogenicity of biopharmaceutical proteins is the challenge that must be solved in the next future.

Aderibigbe made a nice review about in situ gel drug delivery systems designed for nose-to-brain delivery for the treatment of neurological diseases [11]. In situ-based gels are systems that exhibit sol-to-gel transition at the site where they are administered into the body. They were examined according to a classification based on the diseases: in situ-based gels for the delivery of anti-Parkinson’s, anti-migraine, anti-Alzheimer’s, anti-depressant and anti-schizophrenia drugs. This kind of classification is very clear and useful because it allows also a rapid comparison among systems designed for different neurological pathologies. The critical opinion of the authors is reported in what they believe to be the main challenges: neurological diseases have progressive nature and we have insufficient knowledge of them. Future perspectives: there is the need to develop new excipients for nasal in situ gel formulations that reach the clinical stage.

Dalpiaz and Pavan made a review about the way to overcome the active efflux of antiviral drugs, in the case of nose-to-brain delivery [12]. Many antiviral drugs have, in fact, big difficulties in penetrating the brain from the bloodstream because they are substrates of active efflux transporters and this consequently determines a low bioavailability of the drug in the brain. The active transporters are P-glycoproteins. Nasal administration of antiviral drugs is therefore utilized to improve the brain therapies. This paper gives an interesting view of the present situation in this field. Innovative devices, formulations (thermoreversible gels, polymeric micro- and nano-particles, solid lipid microparticles, nanoemulsions), absorption enhancers (chitosan, papaverine), and mucoadhesive agents (chitosan, polyvinilpyrrolidone) are here reported as a way to obtain formulations containing antiviral drugs and suitable for nose-to-brain delivery.

Sonvico et al. made a comprehensive review on surface-modified nanocarriers expressly designed for nose-to-brain delivery [13]. In this review nanomedicine applied to the target of nose-to-brain delivery has been discussed and critically evaluated, considering in particular the influence of physicochemical properties on the characteristics and the “surface chemistry” of the nanoparticles. The authors put in evidence the different points necessary for further development of a medicinal product intended for future nose-to-brain research nanocarrier design. In particular it is pointed out the importance of the design of nanoparticles with biocompatible, biodegradable, “Generally Recognized as Safe” (GRAS) materials. According to the authors’ conclusions, the nasal application of nanocarriers appears to be a way to achieve a non-invasive, efficient, safe, and potentially innovative approach in the treatment of CNS disorders and brain diseases.
